# Pentoxifylline sensitizes human cervical tumor cells to cisplatin-induced apoptosis by suppressing NF-kappa B and decreased cell senescence

**DOI:** 10.1186/1471-2407-11-483

**Published:** 2011-11-10

**Authors:** Georgina Hernandez-Flores, Pablo C Ortiz-Lazareno, Jose Manuel Lerma-Diaz, Jorge R Dominguez-Rodriguez, Luis F Jave-Suarez, Adriana del C Aguilar-Lemarroy, Ruth de Celis-Carrillo, Susana del Toro-Arreola, Yessica C Castellanos-Esparza, Alejandro Bravo-Cuellar

**Affiliations:** 1 División de Inmunología, Centro de Investigación Biomédica de Occidente, Instituto Mexicano del Seguro Social, Guadalajara, Jalisco. México; 2 Laboratorio de Inmunología. Centro Universitario de Ciencias de la Salud, Universidad de Guadalajara, Jalisco. México; 3 Centro Universitario de los Altos, Universidad de Guadalajara, Tepatitlán de Morelos, Jalisco. México

## Abstract

**Background:**

Worldwide, cervical cancer is the second most common causes of cancer in women and represents an important mortality rate. Cisplatin (CIS) is a very important antitumoral agent and can lead tumor cells toward two important cellular states: apoptosis and senescence. In some types of cancers pentoxifylline (PTX) sensitizes these cells to the toxic action of chemotherapeutics drugs such as adriamycin, inducing apoptosis. In the present work, we studied *in vitro *whether PTX alone or in combination with CIS induces apoptosis and/or senescence in cervix cancer HeLa and SiHa cell lines infected with HPV types 16 and 18, respectively, as well as in immortalized keratinocytyes HaCaT cells.

**Methods:**

HeLa (HPV 18+), SiHa (HPV 16+) cervix cancer cells and non-tumorigenic immortalized HaCaT cells (control) were treated with PTX, CIS or both. The cellular toxicity and survival fraction of PTX and CIS were determinate by WST-1 and clonogenic assays respectively. Apoptosis, caspase activation and phosphorylation of ERK1/2, p38, p65 (NF-κB), Bcl-2 and Bcl-XL anti-apoptotic proteins were determinated by flow cytometry. Senescence by microscopy. Phosphorylation of IκBα and IκB total were measured by ELISA. Pro-apoptotic, anti-apoptotic and senescence genes, as well as HPV-E6/7 mRNA expression, were detected by RT-PCR.

**Results:**

Our results show that after 24 hours of incubation PTX *per se *is toxic for cancer cells affecting cell viability and inducing apoptosis. The toxicity in HaCaT cells was minimal. CIS induces apoptosis in HeLa and SiHa cells and its effect was significantly increases when the cells were treated with PTX + CIS. In all studies there was a direct correlation with levels of caspases (-3, -6, -7, -9 and -8) activity and apoptosis. CIS induces important levels of senescence and phosphorylation of ERK1/2, p38, p65/RELA, and IκBα, and decreased the expression of anti-apoptotic protein Bcl-XL. Surprisingly these levels were significantly reduced by PTX in tumor cells, and at the same time, increases the expression of pro-apoptotic genes.

**Conclusion:**

PTX sensitizes cervical cancer cells to CIS-induced apoptosis and decreases the CIS-induced senescence in these cells via inhibition of NF-κB signaling pathway; diminishes expression of antiapoptotic proteins and the activation of caspases.

## Background

Cervical cancer is a major health problem worldwide; it is the second most frequent cause of cancer in women. An estimated 500,000 new cases, were reported in 2008, [1], among which the most important was the presence of human papilloma virus (HPV) infection. High-risk HPV types 16 and 18 are responsible for > 70% of cases of cervix cancer [2,3].

Chemotherapy works in several ways. First, the cells die by apoptosis, which is an irreversible state defined as the genetically programmed cell death, consequently controlled by the balance between proapoptotic and antiapoptotic genes and characterized by cell shrinkage, membrane blebbing, chromatin condensation and nucleosomal DNA fragmentation. Apoptosis is the most convenient manner of tumor cell elimination, because this type of cell death is a final state and the tumor cell does not represent any possible future danger and does not induce inflammation [4-6]. Other tumoral cell response to chemotherapy is the cellular senescence [7]. This cellular state is considered a general biological program of permanent growth arrest and can be induced by telomere shortening (growing old) or by injuries to DNA such as those induced by chemotherapy which do not involve telomere shortening (accelerated senescence). In this state, the tumor cell cannot replicate. This was the reason it was considered originally as a protector mechanism against the development of neoplasia. However, recent data indicates that factors secreted by senescent cells can also alter the microenvironment, and enhance the tumor growth of neighboring tumor cells, indicating that this protective mechanism can act as a double-edged sword. Senescent cells exhibit changes in morphological characteristics such as enlarged and flattened cell shape and increased granularity. This distinction is identifiable with considerable specificity by the detection of β-galactosidase (SA-β-gal) through by X-gal activity staining [8,9].

The antitumor drug Cisplatin (CIS) with clinical and experimental efficiency is employed as a first-line chemotherapeutic modality in the treatment of epithelial malignancies, including lung, ovarian, testicular, cervix cancer and others [10]. From a cell biology viewpoint, the principal mechanism of CIS-induced damage to tumors involves the interaction with DNA and activation of the mitogen-activated protein kinase (MAPK) signaling pathway, which controls a wide spectrum of cellular processes including growth, differentiation and apoptosis [11].

Unfortunately, the chemotherapy's efficiency is so far from satisfactory due to the side effects and to the resistance of tumor cells. Recent publications open the possibility of increasing the efficiency of chemotherapy. Pentoxifylline (PTX), 1-[5-oxohexyl]-3, 7-dimethylxanthine] is a non-specific phosphodiesterase inhibitor that has been routinely employed for circulatory diseases for > 20 years. PTX is a potent inhibitor of tumor necrosis factor-alpha (TNF-α) and the transcription factor NF-κB. In this respect, our group reported that the 100% of lymphoma-bearing mice treated with PTX + adriamycin, an anthracycline, survived for > 1 year after receiving only one half of the therapeutic dosage of adriamycin. Similarly, we also observed that PTX increased the levels of apoptosis generated by adriamycin in fresh leukemic cells of pediatric patients [12-14]. Sensitization of tumor cells to adriamycin by PTX is not tumor type specific. Similar results were observed in hematological and cervical cancer cell lines [15].

The aim of this work was to investigate whether PTX can sensitize cervical cancer cells to apoptosis by means of CIS and modify cellular senescence. Our results indicate that *in vitro*, exposure of cervix tumor cells to PTX-treatment prior to CIS enhances apoptosis levels and reduces cell senescence.

## Methods

### Cell lines

HeLa (HPV-18+) and SiHa (HPV-16+) cervical cancer cell lines and the spontaneously immortalized human epithelial cell line HaCaT (used as non-tumorigenic control cells) were kindly provided by Dr. Boukamp (DKFZ-Heidelberg, Germany). The presence of the human papilloma virus (HPV) type was confirmed by the Linear array^® ^genotyping test (Roche). All of the cell lines were maintained *in vitro *and propagated in Dulbecco's modified Eagle's culture medium (DMEM) supplemented with 10% heat-inactivated fetal bovine serum, 1X L-glutamine (2 mM final concentration) and antibiotics (penicillin/streptomycin). This medium will be referred to as DMEM-S, and was incubated at 37°C in an humidified atmosphere containing 95% air and 5% CO_2_. All of the previously mentioned products were obtained from GIBCO™ Invitrogen Corporation (Carlsbad, CA, USA).

### Drugs and experimental conditions

Cisplatin (CIS) was obtained from PISA Laboratories, México, and stocked at 4°C for < 4 days and adjusted to a desirable concentration with DMEM culture medium immediately prior to utilization. Pentoxifylline (PTX) (Sigma Chemical Co., Saint Louis MO, USA) was dissolved in a sterile saline solution 0.15 M at a concentration of 0.2 M and maintained at 4°C < 4 days.

### Cell culture and *in vitro *treatments

HeLa, SiHa, and HaCaT cells suspended in DMEM-S at concentrations of 1.5 or 2 × 10^6 ^cells/8 mL in exponential phase were seeded in p100 Petri dishes for flow cytometry assays and senescence. For the survival test and for ELISA-determined apoptosis, the cells were cultured in 96-well plates at a concentration of 3 × 10^5 ^cells/well/200 μL (final volume). For clonogenic assays, the cells were seeded at densities of 1 × 10^4 ^cells/2 mL in 6-well plates. In all cases, the cells were cultured overnight at 37°C in a humidified atmosphere containing 5% of CO_2 _and 95% air. The medium was then replaced with DMEM-S. Then the cells were either treated with PTX 8 mM, or with CIS 4 μM or PTX + CIS (final concentrations). These doses of the individual drugs utilized were chosen base on the result of dose-response curves. These doses allow us to observe any further reductions caused by drug combination. The cells were incubated with PTX 1 hours prior to the addition of CIS and 24 hours later the culture cells were harvested. For gene expression study, the cells were incubated with the drugs for only 3 hours.

### Clonogenic cell survival *in vitro*

Cells were assayed for the cytotoxic effects of PTX or CIS or PTX + CIS after cell survival according to the established methods of performing the clonogenic assay. Subconfluent cultures were exposed to the drugs for 6 hours. Then the cells were washed with PBS that was preheated to 37°C, trypsinized and plated in 6-well plates (100 cells/wells). After 15 days of incubation in complete culture medium, the colonies were stained with crystal violet after fixation with formaldehyde and were counted manually. In each case results are expressed as the survival fraction (SF), which was obtained by dividing the number of colonies formed after the treatment/number of cells seeded × PE. Plate efficiency (PE), PE = (*N^o ^*of colonies formed/*N^o ^*of cells seeded) × 100. Colonies (≥ 50 cells) usually appeared in 15 days. The number of colonies on control and drug-treated plates were counted on an inverted-stage microscope at 40-fold magnification. A minimum of 30 colonies/plate was required for an experiment to be considered evaluable for measurement of drug effect [16].

### Drugs interaction analysis

To determine the nature of the interaction between PTX and CIS, the data from the clonogenic assay were analyzed according to Chou and Talalay [17] using CalcuSyn V2.0 software, (Biosoft, Cambridge, UK) [18,19]. For that, the drugs were combined at a constant ratio of PTX and CIS of 2000:1. The interaction of drugs was quantified determining a combination index (CI). CI < or > 1 indicated synergy or antagonism respectively, whereas a CI value of 1 indicates additivity [20].

### WST-1 assay

Cell survival was measured utilizing WST-1/ECS solution (BioVision Research, Mountain View, CA, USA). After 24 hours of incubation 10 μL/well of WST-1/ECS reagent was added and incubated for another 3 hours. Absorbance was measured on a microtiter plate reader (Synergy™ HT Multi-Mode Microplate Reader, Biotek Winooski, VT, USA) at 450 nm. Data are reported in percentage of cell survival as compared with the respectively untreated control group considered as 100%.

### Early apoptosis and caspase activity detection methods

Cellular detection of annexin V, M30 (caspase -3,-6,-7 and-9) and caspase-8 activity was determined by flow cytometry employing the fluorescein isothiocyanate conjugated monoclonal annexin V-FITC apoptosis kit (annexin-V-FLUOS; Roche, Mannheim, Germany), M30 CytoDEATH™ Biotin antibody (Roche Mannheim, Germany), and the fluorescein active caspase-8 staining kit (Abcam, Cambridge, MA) respectively according to the manufacturer instructions. For the three tests at least 20,000 events were analyzed for each sample in an EPICS XL-MCL™ flow cytometer Beckman Coulter model (Fullerton, CA, USA). Data were processed with the System II software package (Beckman Coulter).

### Apoptosis ELISA assays

In normal untreated and treated cell cultures, we determined cytoplasmic histone-associated-DNA-fragments (mono- and oligonucleosomes) spectrophotometrically (420 nm) utilizing Cell Death Detection ELISA^PLUS ^(Roche Mannheim, Germany) according the manufacturer's instruction. Enrichment of mono- and oligonucleosomes released into the cytoplasm was calculated: experimental absorbance/corresponding control absorbance. The results are expressed as the percentage of DNA fragmentation.

### Acridine orange/ethidium bromide staining to detect late apoptosis by Ultraviolet (UV)-microscopy

Briefly, the cells were stained, with ethidium bromide (Sigma Chemical Co. Saint Louis MO, USA) and acridine orange (Sigma Chemical Co. Saint Louis MO, USA) (100 μg/mL each). Two hundred cells were counted and the numbers of each of the following four cellular states were recorded: i) Live cells with normal nuclei (LN), bright green chromatin and organized structure; ii) Apoptotic cells (A) with highly condensed or fragmented bright green-yellow chromatin; iii) Dead cells with normal nuclei (DN), bright red chromatin and organized structure and iv) Dead cells with apoptotic nuclei (DA) and bright orange chromatin, which were highly condensed and fragmented. Apoptotic index (AI): A + DA/LN + A + DN + DA × 100 [21].

### β-galactosidase associated senescence

According to the manufacturer's instructions senescence was determined histochemically in treated and untreated control cells by Senescence Detection Kit (BioVision Mountain View, CA, USA) which detects β-galactosidase activity (SA-β-gal) present in senescence cells. We counted 300 cells of six microscopic fields to determine the percentage of SA-β-gal stained positive cells identified by an intense blue stain in the membrane.

### Protein extraction for IκBα [pS32] and IκBα (total)

15 × 10^6 ^cells were seeded in p150 culture Petri-dishes and treated next day with PTX, CIS and PTX + CIS for 24 hours. After treatment, cells were harvested by scraping and lysed with RIPA buffer (0.5% deoxycholate, 0.5% NP-40, 0.5% SDS, 50 mM Tris pH 7.4 and 100 mM NaCl) containing protein inhibitors. Following sonication (15 pulses, 90% amp), protein extracts were obtained after 30-min incubation at 4°C and 5-min centrifugation at 14,000 rpm/4°C. Protein concentrations were determined using BioRad DC Protein Assay Kit.

### IκBα [pS32] and IκBα (total) ELISA

The levels of I**κ**B**α**[pS32] and IκB**α**(total) protein were determined in HeLa and SiHa treated and untreated control cells employing a commercial ELISA kit (Invitrogen) at 450 nm according to the manufacturer's instructions. The results are expressed as optical density (O.D).

### Bcl-2, Bcl-XL protein expression and phosphorylation state ERK1/2, p38 and p65 by flow cytometry

In normal untreated and treated cell cultures, we determinated the Alexa Fluor^® ^647mouse anti-human Bcl-2 and Alexa Fluor^® ^647 mouse anti human Bcl-XL proteins (Santa Cruz CA) and phosphorylated ERK1/2 (pT202/pY204) PE-Cy™7 mouse anti-human, Alexa Fluor^® ^488 mouse anti-human anti-phospho (P)-p38 (pT180/pY182) and Alexa Fluor^® ^647 mouse anti-human NF-κB p65 (pS529) BD Biosciences by flow cytometry. Cells were resuspended in PBS and stained according to protocol to detecting protein or activation of the phosphorylation state. An appropriate isotype control was utilized in each test to adjust for background fluorescence, and results are reported as Mean fluorescence intensity (MFI). For each sample, at least 20,000 events were acquired in a FACSAria-I cell sorter (BD Biosciences). Data were processed with the FACSDiva software (BD Biosciences).

### Quantitative real time PCR

Total RNA from both types of cells was obtained after 3 hours of incubation using the PureLink™ Micro-to-Midi total RNA purification system (Invitrogen Corporation, Carlsbad, CA, USA). First-strand cDNA was synthesized from 5 μg of total RNA using Superscript™ III First-Strand Synthesis Supermix (Invitrogen Corporation, Carlsbad, CA, USA). Real Time PCR was performed using a LightCycler^® ^2.0 apparatus (Roche Applied Science, Mannheim, Germany) and LightCycler-FastStart DNA Master^PLUS ^SYBR Green I (Roche Applied Science, Mannheim, Germany). Analysis of PCR products was performed using LightCycler^® ^software (Roche Applied Science, Mannheim, Germany). Data are expressed as relative quantities using a reference gene (Protein Ribosomal). Each sample was processed in triplicate to verify the specificity of the amplification reaction. Oligonucleotides (Invitrogen Corporation, Carlsbad, CA, USA) used to amplify human *IκBα, P65/RELA, BAD, BAK, BAX, NOXA, PUMA, P21, P53, P16, MCL-1, BCL-XL*, *CASPASE-3, CASPASE-9, SURVIVIN, E6 *and *E7 (HPV16 *and *HPV18) *and *L32 RIBOSOMAL PROTEIN *are shown in Table 1. Oligonucleotides were designed using the Oligo V6 software. Gene sequences were obtained from the GenBank Nucleotide Database of the National Center for Biotechnology Information http://www.ncbi.nlm.nih.gov.

**Table 1:** Primer pair sequences.

Gene	Primer pair sequences	GenBank Accession No
*IκBα*	5'GGA TAC CTG GAG GAT CAG ATT A 3'	
	5'CCA CCT TAG GGA GTA GTA GAT CAA T 3'	NM001278
*P65/RELA*	5'GCA GGC TCC TGT GCG TGT CT 3'	
	5'GGT GCT CAG GGA TGA CGT AAA G 3'	NM02975
*BAD*	5'CTC CGG AGG ATG AGT GAC GAGT 3'	
	5'ACT TCC GCC CAT ATT CAA GAT 3'	NM004322
*BAK*	5'CGC TTC GTG GTC GAC TTC AT 3'	
	5'AGA AGG CAA AGA CTT CGC TTA 3'	NM001188
*BAX*	5'TTT GCT TCA GGG TTT CAT CC 3'	
	5'CAG TTG AAG TTG CCG TCA GA 3'	NM138764
*NOXA*	5'GAG ATG CCT GGG AAG AAG G 3'	
	5'TCC TGA GCA GAA GAG TTT GGA 3'	NM021127
*PUMA*	5' GAT GGC GGA CGA CCT CAA C 3'	
	5'TGG GAG TCC AGT ATG CTA CAT GGT 3'	NM014417
*P21*	5'CGA CTT TGT CAC CGA GAC AC 3'	
	5'CGT TTT CGA CCC TGA GAG T 3'	NM000389
*P53*	5'CTG AGG TTG GCT CTG ACT GTA CCA CCA TCC 3'	
	5'CTC ATT CAG CTC TCG GAA CAT CTC GAA GCG 3'	NM000546
*P16*	5'CAG TAA CCA TGC CCG CAT AGA T 3'	
	5'TGA AAA GGC AGA AGC GGT GT 3'	NM000077
*MCL-1*	5'CAC GAG ACG GTC TTC CAA GGA TGC T 3'	
	5'CTA GGT TGC TAG GGT GCA ACT CTA GGA 3'	NM021960
*BCL-_XL_*	5'GCA GGC GAC GAG TTT GAA CT 3'	
	5'GTG TCT GGT CAT TTC CGA CTG A 3'	NM138578
*CASPASE 3*	5'ATA CTC CAC AGC ACC TGG TTA T 3'	
	5'AAT GAG AGG GAA ATA CAG TAC CAA 3'	NM004346
*CASPASE 9*	5'GTA CGT TGA GAC CCT GGA CGA C 3'	
	5'GCT GCT AAG AGC CTG TCT GTC ACT 3'	NM001229
*SURVIVIN*	5'TGA GCT GCA GGT TCC TTA TCT G 3'	
	5'GAA TGG CTT TGT GCT TAG TTT T 3'	NM001168
*DIABLO*	5'TGA CTT CAA AAC ACC AAG AGT A 3'	
	5'TTT CTG ACG GAG CTC TTC TA 3'	NM019887
*E6 (HPV 18)*	5'GCG ACC CTA CAA GCT ACC TGA T 3'	
	5*'*GCA CCG CAG GCA CCT TAT TA 3'	X05015
*E7 (HPV 18)*	5'TGT CAC GAG CAA TTA AGC GAC T 3'	
	5'CAC ACAAAG GAC AGG GTG TTC A 3'	X05015
*E6 (HPV 16)*	5'CAG AGC TGC AAA CAA CTA TAC 3'	
	5'AGT GGC TTT TGA CAG TTA ATA C 3'	NC001526
*E7 (HPV 16) *	5'GAC AAG CAG AAC CGG ACA G 3'	
	5'ATT CCT AGT GTG CCC ATT AAC A 3'	NC001526
*L32 RIBOSOMAL *	5'GCA TTG ACA ACA GGG TTC GTA G 3'	
*PROTEIN *	5'ATT TAA ACA GAA AAC GTG CAC A 3'	NM000994

Oligonucleotides were designed using the Oligo v.6 software. Gene sequences were obtained from the GenBank Nucleotide Database of the National Center for Biotechnology Information (http://www.ncbi.nlm.nih.gov).

### Statistical analysis

Results of each experiment represent the means ± standard deviation (SD) of three independent experiments carried out in triplicate. Student's *t*-test was used for statistical analyses a value of *P *< 0.05 was considered significant. For the comparison of gene expression was considered as significant differences values of ≥ 30%. In some cases was calculated the Δ% that represent the percent of increment or diminution in relation to comparative group.

## Results

### Effect of PTX and CIS, alone or in combination on cervix cancer cell line

To evaluate the antiproliferative effects to different schedules of PTX, CIS or PTX + CIS treatments, in a first step we determined the clonogenic assay, which is a proven method to study the chemosensitivity to antitumor drugs. Table 2 shows a clearly dose-response effect in CIS-treated HeLa cultures in which toxicity increased with the dose. Surprisingly, PTX also had cytotoxic effect *per se*, it was also dose-dependant, because with the administered dose of 8 mM, the surviving fraction was approximately 70% lower than that of the untreated control group (*P *< 0.05). The combination of both drugs also shows a similar dose-response effect, reaching near 80% and 100% of toxicity with the two highest doses of PTX 8 and 16 mM and CIS at 4 and 8 μM respectively *P *< 0.05 *vs *untreated control cells. We carried out the same experiments using SiHa cells. The results were similar to those obtained from HeLa cells, but were slightly less efficient. Finally, cells from the non-tumorigenic cell line HaCaT were less sensitive with the different treatments than tumor cells, and highest toxicity was found at the highest dosage.

**Table 2:** Cytotoxic effect of PTX and CIS either alone or in combination on HeLa, SiHa and HaCaT cells.

Agent/combination	Concentration	Surviving fraction	CI
**HeLa cells**			
Pentoxifylline (mM)	2	0.95	
	4	0.44	
	8	0.23	
	16	0.00	
			
Cisplatin (μM)	1	0.97	
	2	0.87	
	4	0.80	
	8	0.08	
			
Pentoxifylline + Cisplatin	2 + 1	0.85	0.795
	4 + 2	0.68	0.605
	8 + 4	0.20	0.974
	16 + 8	0.05	1.766
			
**SiHa cells**			
Pentoxifylline (mM)	2	0.95	
	4	0.54	
	8	0.23	
	16	0.14	
			
Cisplatin (μM)	1	0.94	
	2	0.72	
	4	0.65	
	8	0.10	
			
Pentoxifylline + Cisplatin	2 + 1	0.70	0.753
	4 + 2	0.56	0.380
	8 + 4	0.28	0.970
	16 + 8	0.005	0.840
			
**HaCaT Cells**			
Pentoxifylline (mM)	2	0.90	
	4	0.87	
	8	0.83	
	16	0.75	
			
Cisplatin (μM)	1	0.90	
	2	0.80	
	4	0.78	
	8	0.40	
			
Pentoxifylline + Cisplatin	2 + 1	0.86	1.013
	4 + 2	0.75	1.010
	8 + 4	0.80	1.090
	16 + 8	0.05	0.760

Clonogenic assays were assayed for the cytotoxic effects of PTX or CIS or PTX + CIS. Subconfluent cultures were exposed to the drugs for 6 hours. Then the cells were washed, trypsinized and plated in 6-well plates. After 15 days of incubation, the colonies were stained and counted manually. In each case results are expressed as the surviving fraction. The drug interaction was analyzed according to Chou and Talalay determining a combination index (CI). CI < or > 1 indicated synergy or antagonism respectively, whereas a CI value of 1 indicates additivity. The results represent the mean of three independent experiments carried out in triplicate.

Likewise in Table 2 the interaction of both drugs at different concentrations are shown. We found a synergistic effect in HeLa and SiHa cells with the two lowest doses. With the dose of PTX 8 mM + CIS 4 μM, the drugs interaction can be considered as nearly additive. Finally with highest dose we found a different behavior, so that in HeLa cells we observed a clear antagonic effect. However at highest dose in SiHa cells it was observed a synergic effect. In non-tumorigenic HaCaT cells with highest dose showed a synergic effect and with other doses a nearly additive effect was found.

### Survival Cells *in vitro*

The survival index was determined by WST-1 assay and we found 63.6 ± 2.1% and 57.8 ± 1.0% in HeLa and SiHa cells exclusively treated with PTX respectively. Surprisingly, survival was higher in HeLa cells (98.6 ± 10.5%) and SiHa cells (85.6 ± 9.2%) treated with CIS, than in the groups treated solely with PTX. The most important toxic effect was observed in PTX + CIS groups. Cell survival after treatment was 40.2 ± 1.0% in HeLa and 33.0 ± 1.2% in SiHa cells (*P *< 0.001 *vs *PTX or CIS groups). In contraposition, the addition of CIS to non-tumor HaCaT cells exhibited practically no effect on their survival rate, and the PTX or PTX + CIS treatments slightly decreased the surviving cells (74.1 ± 1.2% and 78.5 ± 1.1% respectively). These data demonstrate that PTX *per se *possesses toxic properties and produces a significant increase of CIS cytotoxicity in human HeLa and SiHa cervical cancer cell lines.

### Early detection of apoptosis in cervix cancer cells

Early-stage of apoptosis was detected by flow cytometry using annexin V and apoptosis progresses by DNA fragments Enzyme-linked immunosorbent assay (ELISA). Table 3 displays results for both tests. All groups showed excellent correlation between both tests. We then observed the same behaviour as in preceding experiment, higher toxicity with the drug combination in comparison with PTX, CIS or control treatments in SiHa cells *P *< 0.001. PTX induces early apoptosis in HeLa cells. In contrast, PTX alone in non-tumor HaCaT cells did not induce early apoptosis in these cells. The most important induction of early apoptosis was observed only in the CIS-treated group (*P *< 0.001 in CIS *vs *PTX and untreated control group). Finally, HaCaT cells cultures in the presence of PTX + CIS exhibited an early apoptosis level comprising that between CIS and untreated control cells.

**Table 3:** Early apoptosis in HeLa, SiHa, or HaCaT cells after *in vitro *exposure to pentoxifylline or cisplatin either alone or in combination.

HeLa		
GROUP	ANNEXIN V	ELISA
	(% mean ± SD)	(% mean ± SD)
**CONTROL**	3.7 ± 1.0	10.0 ± 1.5
**PTX 8 mM**	30.5 ± 1.1 *	30.3 ± 2.3 *
**CIS 4 μM**	10.9 ± 1.4 *	10.0 ± 2.3 *
**PTX + CIS**	25.2 ± 1.3 *	20.1 ± 2.5 *
**SiHa**		
GROUP	ANNEXIN V	ELISA
	(% mean ± SD)	(% mean ± SD)
**CONTROL**	3.7 ± 1.0	10.0 ± 1.5
**PTX 8 mM**	28.4 ± 2.1 *	20.5 ± 1.8 *
**CIS 4 μM**	17.0 ± 0.2 *	17.5 ± 2.7 *
**PTX + CIS**	35.2 ± 1.0 *	30.5 ± 1.5 *
**HaCaT**		
GROUP	ANNEXIN V	ELISA
	(% mean ± SD)	(% mean ± SD)
**CONTROL**	3.8 ± 0.2	10.0 ± 1.5
**PTX 8 mM**	6.0 ± 0.7	10.3 ± 1.8
**CIS 4 μM**	16.3 ± 0.7 *	20.1 ± 2.3 *
**PTX + CIS**	13.5 ± 1.0 *	15.4 ± 1.5 *

Cell cultures were treated with PTX or CIS or their combination, 24 hours later the cells were harvested and early apoptosis was determined by flow cytometry (annexin V-FITC) or ELISA kit (DNA-histone nucleosome). The results represent the mean ± SD of three independent experiments carried out in triplicate. Statistical analysis Student *t *test. (*) *P <*0.001 *vs *untreated control cells.

### PTX sensitizes cervical cancer cells to CIS-induced late apoptosis induced through caspase activation

**Figure 1:**
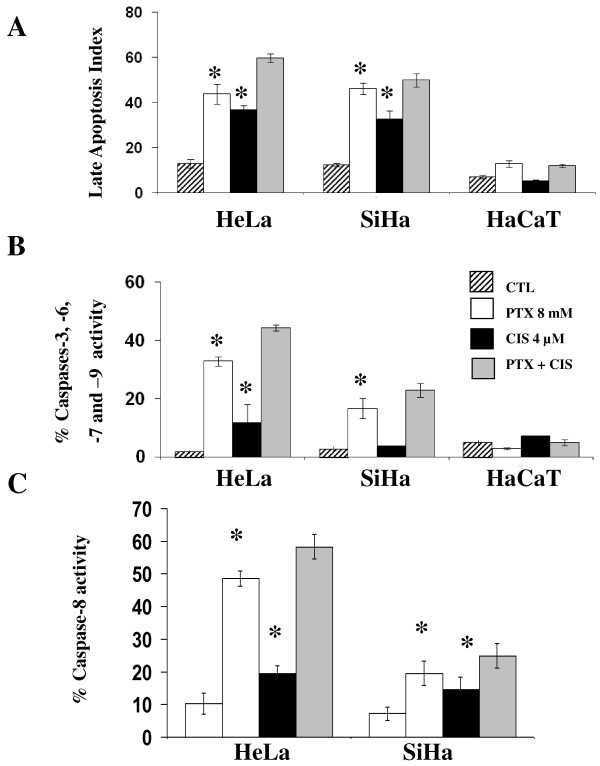
**Determination of late apoptosis and caspase activity of HeLa, SiHa and HaCaT cells after *in vitro *treatment with pentoxifylline or cisplatin either alone or in combinations**. 24 hours later the cells were harvested and late apoptosis was determined by UV light microscopy using ethidium bromide and acridine orange stains, the results represent late apoptosis index (Figure 1A). Caspases-3, -6, - 7, -9 and -8 activation was determined by flow cytometry, the results represent the percentage of caspase activity (Figure 1B and 1C respectively). The results represent the mean ± SD of three independent experiments carried out in triplicate. Statistical analysis, Student's *t *test. (*) *P *< 0.001 *vs *CTL. (♦) *P *< 0.001 *vs *CIS.

Apoptosis can be reversible in the first steps; for this reason we also determined late apoptosis by epifluorescence. Figure 1A shows that in all cases in untreated control groups, the apoptotic index was ≤ 13. In contrast, in all treated groups, important levels of apoptosis were detected, because when HeLa and SiHa tumor cells were treated with PTX alone, the apoptotic indexes were 43.8 ± 4.4 and 46.2 ± 2.4 respectively (*P *< 0.001 *vs *untreated group). The apoptotic index induced by CIS alone in HeLa (36.8 ± 3.8) and SiHa cells (32.6 ± 2.9) were slightly lower than those obtained with PTX alone, but higher than those of untreated tumor cells, respectively (*P *< 0.001). Interestingly, the most important indexes of apoptosis were obtained with the combination of PTX + CIS reaching for HeLa an apoptotic index of 59.8 ± 1.8 and for SiHa cells 47.2 ± 2.9 (*P *< 0.001 *vs *untreated cells). In contrast, in HaCaT cells treated with PTX, CIS or its combination, apoptotic indexes were similar to those untreated cells.

It is well known that caspases play a central role in apoptosis, because that we studied the caspases activation pathways. Participation of caspases-3,-6,-7 and -9 was determined by flow cytometry using M30 antibody. In Figure 1B it can be observed that the three untreated cells lines displayed minimal caspases activity (≤ 5.0%). PTX culture exposure increases by 17.2 times the percentage of M30 positive cells in HeLa and by 5.8 times in SiHa (*P *< 0.001 *vs *untreated cells). CIS induces an increase of caspase activation in HeLa cells of 6.2 times higher than in untreated cells and had no effect in SiHa cells. However, in PTX + CIS-treated cells, we found a clear additive effects in both cervical tumor cell lines, observing a increment of positive cells to caspase activity of 23.3 and 6.5 times higher, respectively, than of untreated control cells (*P *< 0.001 *vs *untreated cells).

In Figure 1C, it can observe that untreated group of HeLa and SiHa cells displayed minimal caspase-8 activity, but when these cells were treated with PTX, we found increments of caspase-8 activity to be 4.2 and 2.7 fold higher in HeLa and SiHa cells, respectively (*P <*0.001 *vs *untreated cells), also CIS alone induces an increase of caspase-8 activity but lower that the increment induced by PTX (HeLa 1.71 and SiHa 1.9 times higher than corresponding untreated cells). The higher increments on caspase-8 activity was found in PTX + CIS treated groups were this treatment HeLa and SiHa reached increments of 5.1 and 3.2 times higher than the CIS treated group (*P *< 0.001).

### PTX decreases CIS-induced senescence

**Figure 2:**
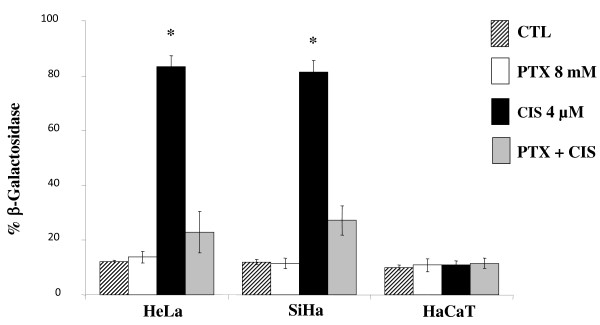
**Determination of β-galactosidase-associated senescence of HeLa, SiHa and HaCaT cells after *in vitro *treatment with PTX or CIS either alone or in combinations**. 24 hours later the cells were harvested and senescence was determined by histochemistry using senescence detection kit (BioVision Mountain View, CA, USA). The results represent the mean ± SD of three independent experiments carried out in triplicate. Statistical analysis, Student's *t *test. (*) *P *< 0.001 *vs *CTL. (♦) *P *< 0.001 *vs *CIS.

Senescence was measured by determination of the β-galactosidase. In all untreated cell lines studied, the percentage of senescence was minimum (≤ 12.1 ± 0.6%) (Figure 2). It is noteworthy that PTX does not induce senescence in all cell lines. In opposite fashion, CIS induced high levels of senescence in comparison with untreated control cells: 6.9 times higher in HeLa (83.3 ± 4.1%) and in SiHa cells (81.5 ± 4%), and in both cases *P *< 0.001 *vs *the untreated control group. CIS does not modify the percentage of senescence in HaCaT cells. In HeLa and SiHa cells treated with PTX + CIS the percentage of SA-β-Gal(+) was significantly lower (22.9 ± 7.5% and 27.2 ± 5.4%, respectively) which represents a 3.6 and 3-times lower diminution in relationship to senescence induced by CIS alone (*P *< 0.001).

### Total IκBα and IκBα Phosphorylated in serine 32 (IκB-pS32)

**Figure 3:**
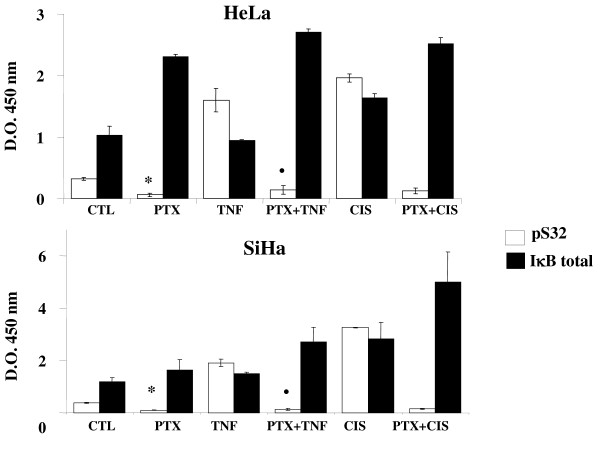
**Phosphorylation of the IκBα [pS32] and IκBα (Total) by ELISA kit of HeLa and SiHa cells after *in vitro *treatment with pentoxifylline or cisplatin either alone or in combination**. 24 hours later the cells were harvested and the phosphorylation of the IκBα [pS32] and IκBα (Total) was determined by commercial ELISA kit (Invitrogen). The results represent the mean ± SD of three independent experiments carried out in triplicate. Statistical analysis Student's *t *test. (*) *P <*0.001 *vs *CTL. (•) *P <*0.001 *vs *TNF-α. (♦) *P <*0.001 *vs *CIS.

As a central point, in this set of experiments we quantified the total IκBα and the phosphorylated form. Our observations in Figure 3c learly showed that with both cervical tumor cells, all treatments increased total IκBα in relationship to the phosphorylated form IκBα from untreated control groups, respectively, except in TNF-α treated cultures (*P *< 0.001). In all PTX-treated groups, the phosphorylated form with both tumor cell lines was diminished in comparison with the respective untreated control groups (*P *< 0.001). In contraposition, and again in both tumor cell lines treated with TNF-α or CIS, the phosphorylated fraction was drastically incremented (*P *< 0.001 *vs *CTL, PTX). Likewise the PTX diminished the phosphorylation of IκBα induced by CIS or TNF-α *P *< 0.001.

### Phosphorylated ERK1/2, p38 and p65 determination

**Figure 4:**
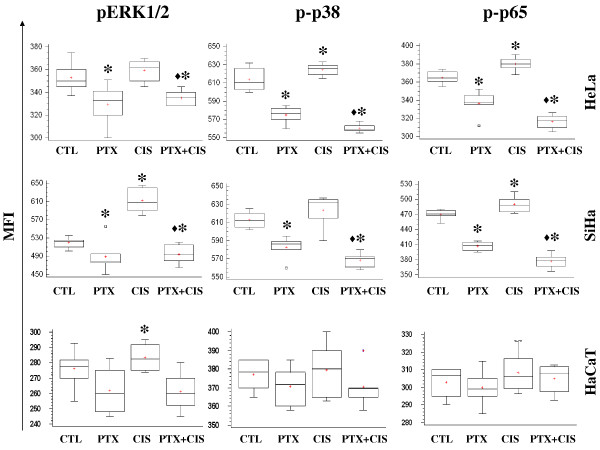
**Determination of phosphorylated ERK 1/2, p38, and p65 in HeLa, SiHa and HaCaT cell treatment with pentoxifylline or cisplatin either alone or in combination**. 24 hours later the cells were harvested and the phosphorylated ERK1/2, p38 and p65 proteins were determined by flow cytometry. A total of 20,000 events were registered in each test. The results represent the mean ± SD of 3 independent experiments carried out in triplicate. (*) *P <*0.001 *vs *untreated control cells. (♦) = *P <*0.001 *vs *CIS.

On the other hand when the cells are stressed by chemotherapy the phosphorylation of ERK1/2, p38 and p65 (NF-κB subunit) proteins play a central role in cell proliferation, differentiation, and survival. Under our experimental conditions, these proteins were determined by flow cytometry and the results are reported as Mean fluorescence intensity (MFI). In Figure 4 we can observe that pERK1/2 expression in HeLa, SiHa and HaCaT decreased in cells treated with PTX compared with untreated group (*P *< 0.001). In SiHa cells, CIS increased phosphorylation of ERK1/2 and PTX + CIS-treated group decreased this phosphorylation (*P *< 0.001).

Expression of phosphorylated p38 in HeLa and SiHa tumor cells was inhibited significantly in the cells harvested, from PTX alone and PTX + CIS treated cultures (*P *< 0.001 *vs *CIS or untreated cells), while treatment with CIS alone showed an MFI similar to that of the respective untreated group in SiHa cell and an increased in HeLa cells (*P *< 0.001). HaCaT cells did not differ significantly among all groups.

We also determined the phosphorylation of p65 (NF-κB subunit). The behavior of HeLa and SiHa cells was similar to that in previous experiments because PTX alone or in combination with CIS significantly inhibited the phosphorylation of p65 (*P *< 0.001) in comparison with that of untreated cells and the CIS group. In HeLa and SiHa cells, CIS increased p65 phosphorylation in comparison with that untreated cells (*P *< 0.001). Finally HaCaT cells did not modify the expression of phosphorylated p65 protein with any treatment. All groups showed similar values to untreated control HaCaT cells.

### PTX decreased Bcl-2 and Bcl-XL anti-apoptotic proteins

**Figure 5:**
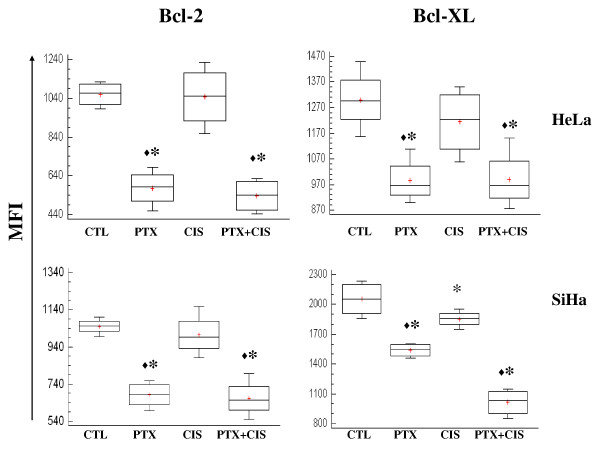
**Determination of Bcl-2 and Bcl-XL anti-apoptotic proteins in cervical tumor cells treated with PTX or CIS either alone or in combination**. 24 hours later the cells were harvested and the proteins expression were determined by flow cytometry. A total of 20,000 events were registered in each test. The results represent the mean ± SD of 3 independent experiments carried out in triplicate. (*) = *P *< 0.01 *vs *CTL. (♦) *P *< 0.05 *vs *CIS.

NF-κB pathway regulates the anti-apoptotic proteins Bcl-2 and Bcl-XL. The elevated levels of these proteins confer chemoresistance. Participation of Bcl-2 and Bcl-XL was determinated by flow cytometry. Figure 5 shows that PTX is able to markedly down-regulate the expression of Bcl-2 and Bcl-XL proteins in both HeLa and SiHa cells as compared with untreated cells (*P *< 0.001). We observed a decreased Bcl-XL protein expression in SiHa cells treated with CIS in comparison to untreated cells (*P *< 0.05). The group treated with a combination treatment of PTX + CIS, a marked decrease in Bcl-2 and Bcl-XL was detected compared with untreated cells or treated with CIS (*P *< 0.05).

### PTX, CIS or PTX + CIS modifies caspase, proapoptotic and antiapoptotic, senescence and NF-κB related gene expression

**Figure 6:**
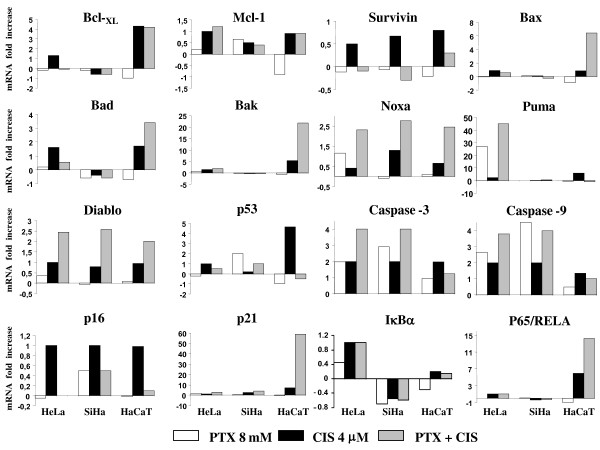
**Changes in the expression of caspases, senescence, NF-κB, pro- and antiapoptotic-related genes after *in vitro *exposure to pentoxifylline or cisplatin either alone or in combination**. The gene expressions were determined by real-time quantitative PCR. The data are expressed as mRNA fold-increase using mRNA ribosomal as a reference gene. Experiments were conducted in triplicates and repeated three times. In all cases, SD was not > 0.08.

Real time-PCR was employed to determine mRNA expression (Figure 6). In PTX-treated HeLa cells, we found 1.3 to 3 fold up-regulation of IκBα, P65/RELA, CASPASES-3 and -9, P21, BAK and NOXA. In PUMA gene expression, we found a > 28 fold up-regulation with PTX. When the cells were treated with CIS, we observed 1.3 to 3 fold up-regulation of P53, P16, BAX, BAD, BAK, NOXA, CASPASES-3, -9, IκBα, P65/RELA, BCL-_XL _and MCL-1, P21 and PUMA. In PTX + CIS treated HeLa cells we observed 1.3 to 3 fold up-regulation of IκBα, P65/RELA, P53, BAK, BAX, BAD, P16 and MCL-1 up-regulation of > 3-fold in CASPASES-3, -9, NOXA and P21. However, the up-regulation was greater in PUMA (45 fold). PTX-treated SiHa cells demonstrate 1.4- to 3-fold up-regulation in CASPASE-3, P53, P16 and P21 genes and an increase of > 3-fold in CASPASE-9. In the same manner, CIS induced a 1.3- to 3-fold up-regulation of CASPASES-3, -9, P21, NOXA, P16 and DIABLO. When SiHa cells were treated with PTX + CIS, mRNA expression levels of P53 and PUMA, and P16 were 1.3- to 3-fold up-regulated, while in CASPASES-3, -9, NOXA and P21 we found > 3-fold up-regulation. Finally, in CIS-treated HaCaT cells, we found 1.3- to 3-fold up-regulation in CASPASES-3, -9, BAX, BAD, NOXA, P16 and MCL-1 and one of > 5-fold in P65, P53, PUMA, BAK, P21 and BCL-_XL_. When the HaCaT cells were treated with PTX + CIS, we found a 1- to 3-fold increase in MCL-1 gene and > 2.5-fold in NOXA, BAD, P65/RELA, PUMA and BCL-_XL_. Moreover, we observed > 20-fold increase in BAX and a 60-fold in P21 genes; in contrast, P53 was inhibited 1.3-fold. With these treatment schedules, the data in general suggested that activation is in favor of genes with proapoptotic activity in PTX + CIS-treated HeLa and SiHa cancer cells.

### Expression of E6 and E7 mRNA from HPV 16 and 18 on HeLa and SiHa cells respectively, determined by real-time PCR

E6 and E7 play a key role in cervical carcinogenesis. We analyzed, in human cervical carcinoma cell line HeLa and SiHa, the gene expression of the viral oncogenic E6 and E7. This set of experiments was performed under the same experimental conditions, and the results are reported as the Δ% of the values obtained, taking as 100% the expression of the constitutive ribosomal mRNA. In the case of HPV-18 positive HeLa cells, the expression of E6-E7 mRNA was modified only in the PTX + CIS-treated group, which achieved an increase of Δ% = 22. For the case of E7 mRNA expression, we observed in the same line a slight decrease (Δ% ≤ 12% for PTX- and CIS-treated groups) and no variation was observed in PTX + CIS-treated group. The mRNA expression of E6 and E7 in SiHa cells (HPV-16+) was significantly inhibited in relation to untreated control group, because for E6 mRNA expression was Δ% = -48, -59 and -58% from culture cells treaded with PTX, CIS and PTX + CIS, respectively, while for E7 mRNA expression, Δ% was -42, -65 and -60% respectively.

## Discussion

In the present work, we found good correlation between survival and different apoptotic assays. Surprisingly, PTX *per se *results toxic for HeLa and SiHa tumor cells and sensitizes these to the toxic action of CIS, increasing apoptosis and simultaneously reducing senescence. It is also noteworthy that as an advantage, PTX is more toxic than CIS in cancer cells and was practically not toxic for non-tumorigenic HaCaT keratinocytes.

We detected early and late apoptosis because in the first steps apoptosis can be reversible [22]. The UV light microscopy test allowed us to appreciate a definitive status. The observation that non-tumorigenic HaCaT cells are less sensitive to different treatments is probably due to the fact that the rate of multiplication and metabolism is slower in HaCaT cells than in tumor cells.

These results are in agreement with other published data reporting that PTX sensitizes *in vivo *and *in vitro *cancer cells to chemotherapy, particularly to adriamycin [12]. Within this context, we previously reported that the PTX is able to sensitize lymphoma and leukemic cancer cells to apoptosis by adriamycin or perillyl alcohol [13]. Similar results have been reported with radiotherapy [23]. The observations of the present work are in agreement with recent data in which our group demonstrated that PTX increases apoptosis and inhibits senescence in HeLa and SiHa Cells treated with adriamycin, an anthracycline used also against cervical cancer [15]. The present results are important because CIS is the first drug of election in the treatment of cervical cancer. Additionally to published data, the results of the present work strongly suggest that the cytotoxicity of PTX is not limited to one type of tumor cells or to chemotherapeutic drugs, incrementing its potential utilization in Oncology.

The low toxicity showed by CIS in survival test may be explained because CIS induces senescence. Senescence originally was considered to be a tumor-suppressor mechanism [24,25]. However its role in Oncology is not clear because senescent cells though they cannot replicate, continue releasing growth factors, enzymes and other products that under certain conditions promote tumor growth [9,26]. It is very interesting that PTX does not induce senescence, and strongly decreases the senescence induced by CIS. The importance of these observations is that an antitumoral treatment that induces principally apoptosis rather than senescence is preferable in cancer cells.

Different mechanisms can explain our observations. PTX also has antimetastatic activity [27] and arrests the cell cycle in the G2/M, in which the tumors are more sensitive to the toxic effects of some chemotherapeutic and radiotherapeutic agents [28,29]. PTX has been linked as well to the activation of caspase [12,30]. In this study, an important activity of caspase (-3, -6 -7 -9 and -8) was detected in HeLa and SiHa cells treated with PTX or PTX + CIS and, in minor degree, with CIS. In addition, this caspase activity is directly proportional to the level of apoptosis confirming its participation. In SiHa cells treated with CIS alone, we observed low caspase activity. In this regard, it has been reported that CIS may also exert its apoptotic activity by caspase-independent pathways [31].

PTX is a strong inhibitor of phosphodiesterase activity. In murine lymphoma and U937 human monocyte cell line, it also prevents activation NF-κB in these cells [12] by inhibition of the phosphorylation of serine 32 in IκB complex. Thus preventing TNF-α secretion and expression of certain antiapoptotic genes that possess antioxidant activity [32]. Contrariwise, CIS promotes the formation of reactive oxygen species (ROS), which provoke apoptosis or senescence [33].

We also studied the phosphorylation of different proteins that are important for proliferation, differentiation, cell survival, apoptosis and senescence such as ERK1/2 and p38 from the family of mitogen activated protein kinases (MAPKs) and phosphorylation of the p65 subunit of NF-κB and related IκB proteins. Induction of death by CIS has been associated with increase in p38 and ERK1/2 activity [11,34]. We observed this activity in SiHa and HeLa cells, but it has been demonstrated that ERK1/2 activity induced by CIS can cause resistance in SiHa cells [35], gastric cancer cells [36], and human myeloid leukemic cells [37]. PTX decrease ERK1/2 phosphorylation in SiHa cells, this disrupts resistance to CIS, because when we utilized PTX, apoptosis was higher than in CIS-treated cells. Is it noteworthy that, PTX decreased the phosphorylation of p65 and IκBα (S32), thus resulting in the inhibition of nuclear translocation of NF-κB and avoiding the cell survival and resistance observed in CIS-treated cells [38-40]. NF-κB can activate different genes related with the cell survival such as Bcl-2 and Bcl-XL [41]. It's important to stress that PTX by itself or in combination with CIS disrupt the NF-κB pathway. We observe an inhibition of phosphorylation the IκBα, p65 and decrease the levels of anti-apoptotic proteins Bcl-2 and Bcl-XL in HeLa and SiHa cells. This is important because these antiapoptotic proteins confer resistance to several chemotherapeutic agents including CIS, gemcitabine, vincristine, etoposide, doxorubicin, and paclitaxel [42].

In our study, PTX significantly disrupted the CIS resistance in HeLa and SiHa cell by blocking the NF-κB mediated survival pathway. PTX possesses an additive effect with CIS (8 mM + 4 μM respectively); the combined usage of these two drugs promotes apoptosis of cervical tumor cells and at the same time impairs senescence.

Our results suggest that PTX action on NF-κB, ERK1/2, p38, Bcl-2 and Bcl-XL proteins and caspases can explain the fact that it does not induce senescence, but does increase apoptosis in HeLa and SiHa cells. In addition, when we employed PTX in combination with CIS, it impaired CIS-induced senescence and increased the sensitivity of these cervix cancer cells to this drug. Therefore, we think that PTX could be used to abrogate NF-κB-induced resistance mechanisms without severe systemic toxicity. Thus, the use of PTX with other chemotherapeutic agents such as CIS may lead to more efficient cervical cancer cell elimination.

Moreover, a gene expression analysis to study the antitumoral effects of drugs is critical in order to identify the potential PTX + CIS-specific genetic targets involved. Employing an RT-PCR assay, we studied the mRNA expression of genes related NF-κB pathway, apoptosis and senescence. In general, we observed in HeLa and SiHa cervix cancer cells an up-regulation of some proapoptotic genes after PTX + CIS treatment, including the DIABLO, NOXA, PUMA, CASPASES-3 and -9 genes, which are implicated in the mitochondrial pathway of apoptosis [43]. It is noteworthy that treatment with CIS induces the expression of anti-apoptotic gene, SURVIVIN. These phenomena have been reported as another cause of tumor-cell resistance to chemotherapy [44,45]. Up-regulation of SURVIVIN is also present in senescent tumor cells. To the contrary, treatment with PTX alone in all experimental groups, down-regulated the expression of SURVIVIN gene. These results show that PTX can overcome one of the survival strategies used by the cancer cells in response to chemotherapeutic agents. The Bcl-2 family genes protect the cells of CIS-induced apoptosis [46,47]. This fact contributes to the explanation of all our results because we found that some survival genes are down-regulated by PTX, as it the case with BCL-_XL_. The strongly over-expression of some pro-apoptotic genes likes PUMA (4500%), tip the balance in favor of apoptosis. CIS administration paradoxically leads to an antiapoptotic effect of p53 pathway, which induces tumor cell resistance to CIS [48,49]. In our work, we demonstrated that PTX counteracts this effect by promoting apoptosis in HeLa and SiHa cells, as confirmed by the over-expression of PUMA, NOXA and P21 genes which are regulated by p53 [50]. This does not exclude the existence of other p53-independent pathways for induction of apoptosis, because we found a slight over-expression of P53 compared with the high over-expression of NOXA, PUMA and P21 genes [51-53]. It is important to remark that these results together agree with the direct determination of the most important proteins related with apoptosis and the cell survival under our experimental conditions. The senescence-associated P16 gene, exhibits a different behaviour between two cancer cervix lines. CIS induced up-regulation of the P16 gene in HeLa and SiHa cancer cells, is incomplete accordance to the senescence levels observed in β-galactosidase assay in these cells.

With regard to IκBα and P65/RELA genes, related to transcription factor NF-κB, IκBα and P65 expression, were down-regulated or remained unchanged with all treatments in SiHa cells, suggesting a diminution of the availability of these factors, which facilitate cell apoptosis. However, in the three treated groups of HeLa cells, we observed an up-regulation of IκBα and P65/RELA genes strictly that was comparable between these genes suggesting an equal balance of both factors.

In the non-tumorigenic line HaCaT we observed a different behaviour in comparison with cervical tumor cells. In general, we noted an important activation of genes with proapoptotic activity, including BAB, BAX, NOXA and P21 (CIS and PTX + CIS), as well as in PTX groups for CASPASE-3 gene. However, despite of the up-regulation of several proapoptotic genes, apoptosis levels were low and cell viability was not affected, suggesting that the rate of multiplication displays an important effect in the action of the assayed drugs. In this respect, is also important to mention that P65 is up-regulated > 7-fold and BCL-_XL _5-fold, and we found no important levels of apoptosis.

Because expression of mRNA E6/E7 genes appear to play a key role in cervical cancer development, we conducted an analysis in human cervical carcinoma SiHa and HeLa cell line. We observed a decrease in the expression of E6 and E7 genes only in SiHa cells, treated with the different drugs, although in HeLa cells we observed no effect on these genes. In both cancer cell lines, we observed induction apoptosis and sensibilization by PTX. This indicates that several mechanisms of resistance and susceptibility to antitumoral drug could be implicated, such as the HPV types and their interactions with the cells.

The choice between survival, senescence or apoptosis, is a very complex process [54,55]. Rather than the action of a single gene or molecules, the final balance between activation or not of these genes and molecules determines whether or not a cell undergoes apoptosis. In this study, we observed an overall balance in favor of the apoptotic process in HeLa and SiHa cancer cells treated with PTX and/or CIS.

## Conclusions

Our observations show that PTX possesses antitumor activity and inhibits cisplatin-induced senescence. The novel combination of PTX + CIS which sensitizes HeLa and SiHa cancer cells, to the toxic effect of CIS without affecting the viability of non-tumorigenic cell line, may be a promising approach to the treatment of patients suffering from cervix cancer.

## List of abbreviations

PTX: pentoxifylline; CIS: cisplatin; HPV: human papilloma virus; NF-κB: nuclear factor kappa-B; SA-β-gal: β-galactosidase activity; CI: combination index;

## Competing interests

The authors declare that they have no competing interests.

## Authors' contributions

GHF, ABC, PCOL designed and performed the research, analyzed the data and drafted the manuscript; JMLD, JRDR, YCC and RCC performed some of the research and analyzed the data, AAL, LFJS and STA performed molecular study. All the authors read and approved the final manuscript

## Pre-publication history

The pre-publication history for this paper can be accessed here:

http://www.biomedcentral.com/1471-2407/11/483/prepub
